# Evaluation of Peri-Implant Bone Repair in Ovariectomized Rats Submitted to the Implant Placement Functionalized with Anti-Sclerostin

**DOI:** 10.3390/bioengineering12040358

**Published:** 2025-03-30

**Authors:** Pedro Henrique Silva Gomes-Ferreira, Paula Buzo Frigério, Nathália Dantas Duarte, Juliana de Moura, Naara Gabriela Monteiro, André Luis da Silva Fabris, Roberta Okamoto

**Affiliations:** 1 Department of Dentistry, Ourinhos University Center (UNIFIO), Ourinhos 19909-100, SP, Brazil; pedroferreirabmf@gmail.com; 2 Department of Diagnosis and Surgery, Araçatuba School of Dentistry, São Paulo State University (UNESP), Araçatuba 16015-050, SP, Brazil; duarte-nathalia@hotmail.com (N.D.D.); juliana.moura032@outlook.com (J.d.M.); naaragmonteiro@gmail.com (N.G.M.); a.fabris@unesp.br (A.L.d.S.F.); 3 Department of Basic Sciences, Araçatuba School of Dentistry, São Paulo State University (UNESP), Araçatuba 16015-050, SP, Brazil; roberta.okamoto@unesp.br

**Keywords:** bone, dental implants, osseointegration, osteoporosis, rat, tibia

## Abstract

(1) Background: The challenges in Implantology involve the development of alternative methods to enhance bone repair in patients with systemic conditions, such as osteoporosis. This study aimed to evaluate the effect of a local anti-sclerostin monoclonal antibody (Scl-Ab) on the functionalization of titanium implant surfaces through a dip-coating technique in peri-implant bone repair. (2) Methods: A total of 32 female rats were separated into four groups (n = 8): SHAM NT (Sham surgery), OVX NT (ovariectomy), SHAM Scl-Ab (SHAM; implants functionalized with Scl-Ab), and OVX Scl-Ab (OVX; implants functionalized with Scl-Ab). Implant surgery was executed 30 days after ovariectomy, and the rats were euthanized 28 days postoperatively. The right tibia was used for removal torque and RT-PCR, while the left tibia was collected for micro-CT and laser confocal microscopy. (3) Results: Functionalization with Scl-Ab significantly increased the gene expression of bone markers, especially ALP, in the SHAM Scl-Ab group compared to the other groups (*p* < 0.05). (4) Conclusions: Some parameters of this study indicate that implants functionalized with anti-sclerostin bone anabolic drug enhance peri-implant bone repair, especially in healthy rats. However, more studies must be carried out to confirm the therapeutic benefits of this approach.

## 1. Introduction

Osteoporosis is a systemic disease that leads to the progressive deterioration of bone microarchitecture due to an imbalance in bone tissue remodeling [1]. This condition is recognized as a major health concern for women, particularly postmenopausal women, primarily due to estrogen deficiency [2,3].

Pharmacological treatments for osteoporosis are categorized into anabolic and antiresorptive agents [4]. Bisphosphonates, selective receptor modulators of estrogen (SERMs), and Denosumab (anti-RANKL antibody) represent antiresorptive agents [5]. At the same time, parathyroid hormone (PTH) and Romosozumab (anti-sclerostin antibody) belong to the anabolic group, which stimulates new bone formation [6,7]. Although antiresorptive drugs, particularly bisphosphonates, are the most commonly prescribed for osteoporosis, their use in dentistry raises concerns due to the risk of medication-related osteonecrosis of the jaws [8,9].

The anti-sclerostin monoclonal antibody (Scl-Ab), such as Romosozumab, is a natural agonist of the Wnt/β-catenin pathway and increases bone mineral density and at the same time decreases bone reabsorption during the first months of the drug administration [7,10]. Some clinical studies identified the potential side effects associated with Romosozumab: inflammatory reactions, arthralgia, myalgia, and the increased risk of cardiovascular events, such as myocardial infarction and cerebrovascular accident due to possible vascular calcification [11,12]. Therefore, this medication is indicated for the treatment of severe osteoporosis in postmenopausal women with a high risk of fracture and no cardiovascular events [7].

The impact of estrogen deficiency on peri-implant bone healing has already been demonstrated in preclinical studies with ovariectomized rats [13,14,15,16]. Therefore, due to the action of Scl-Ab as a bone anabolic, this study hypothesizes it may be a potential approach to improve osseointegration in patients with osteoporosis [17,18,19]. For this reason, this work aims to evaluate the effect of a local anti-sclerostin monoclonal antibody (Scl-Ab) on the functionalization of titanium implant surfaces through a dip-coating technique in peri-implant bone repair.

## 2. Materials and Methods

### 2.1. Ethics and Calculation of Sample Size

The Ethics Committee on the Use of Animals from Araçatuba School of Dentistry of São Paulo State University (FOA-UNESP) approved this study, protocol 2021-0318. All the reported data agree with the Animal Research: Reporting of In Vivo Experiments (ARRIVE Guidelines 2.0) [20]. All experiments were performed under blinding and calibration. The sample size for each group in the present study was determined using a power test calculator (OpenEpi, Version 3.01, Open-Source Calculator) based on previously published results [19]. The calculation used means 3.06 and 4.898 and standard deviations of 0.26 and 0.024, with a significance level of 5% and a power of 95% in a one-tailed hypothesis test. Therefore, 8 rats were included per group, totaling 32 female four-month-old Wistar rats (Rattus norvegicus albinus) weighing approximately 300 g. The animals were kept at the Central Bioterium in a climate-controlled environment with a twelve-hour light and dark cycle. All the rats were kept in cages with water and regular feed (Nuvilab, Curitiba, PR, Brazil).

### 2.2. Experimental Design

The animals were randomized using a computer-generated list created in Stata 9.0 (StataCorp, College Station, TX, USA). The rats were submitted to SHAM surgery or bilateral ovariectomy (Day 0). The analyses proposed for this study were divided into calcified sections—micro-CT and laser confocal microscopy (left tibias); biomechanical analysis—removal torque and molecular biology—RT-PCR (right tibias). The non-treatment groups, SHAM NT and OVX NT, were considered the positive and negative control, respectively, for comparison with the other groups. These two groups received conventional implants and were used to compare with the groups treated with local Scl-Ab. The SHAM Scl-Ab group consisted of healthy animals that received Scl-Ab functionalized implants.

**Figure 1:**
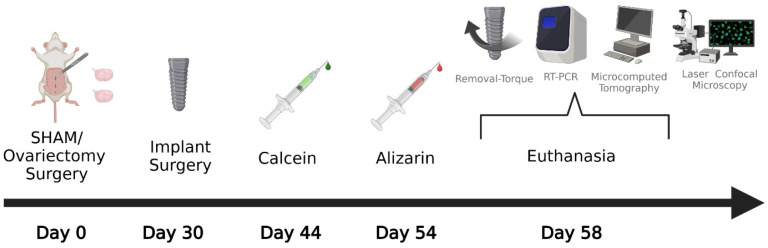
Study design diagram. Created in https://BioRender.com, accessed on 12 February 2025.

In contrast, the OVX Scl-Ab group received the same functionalized implants but had previously undergone ovariectomy. On day thirty (Day 30), the implants were placed in the tibias of these animals. Fourteen days after the implant placement surgeries, all experimental groups received an intramuscular injection of 20 mg/kg calcein (Sigma-Aldrich, St. Louis, MO, USA) (Day 44). Ten days later, the same animals received an intramuscular injection of 20 mg/kg alizarin (Sigma-Aldrich, St. Louis, MO, USA) (Day 54). Twenty-eight days after implant placement (Day 58), the animals were anesthetized, and the implants were removed using a digital torque wrench to obtain removal torque data from the right tibias. Immediately after implant removal, the bone tissue in contact with the implant threads was collected for RT-PCR. The left tibias were reserved for micro-CT and laser confocal microscopy, as shown in the study diagram in Figure 1.

### 2.3. Estrous Cycle

To ensure that the rats selected for the experiments were cycling normally, they were introduced to individual cages, and one or two drops of saline solution were placed into the vagina daily. The solution was aspirated and placed on a histological slide for reading in a microscopic optical to observe the phases of the cycle [21]. The rats were used after obtaining two regular estrous cycles. This procedure was performed again to validate the bilateral ovariectomy surgery, indicated by the constant diestrus phase.

### 2.4. Ovariectomy

All the surgical procedures were performed at the Laboratory for Study of Mineralized Tissue (LSMT) at the Department of Basic Sciences of FOA-UNESP. The animals were fasted for eight hours before the ovariectomy. The rats were anesthetized with 5 mg/kg of xylazine (Fort Dodge Saúde Animal Ltda., Campinas, SP, Brazil) and 50 mg/kg of ketamine (Fort Dodge Saúde Animal Ltda., Campinas, SP, Brazil). The incision was made on the flanks using blade n^o^ 15 (Feather Industries, Tokyo, Japan) mounted on a scalpel handle n^o^ 3 (Hu-Friedy, Frankfurt, Germany) to access the abdominal cavity. The uterine tubes were ligated with an absorbable suture (Polyglactin 910 4.0, Ethicon^®^; Johnson & Johnson, São José dos Campos, SP, Brazil), and the ovaries were removed with scissors. Once finished, the surgical wound was closed with simple sutures (Polyglactin 910 4.0, Ethicon^®^; Johnson & Johnson, São José dos Campos, SP, Brazil). The SHAM group rats underwent the same procedure, but only surgical exposure of the ovaries was performed without removal.

### 2.5. Functionalization of the Implants

Sixty-four commercially pure grade IV titanium implants (1.5 mm × 3.5 mm; Titaniumfix^®^, São José dos Campos, SP, Brazil) with a surface treated with double acid enticing using sulfuric acid, nitric, and hydrofluoric were functionalized with Scl-Ab (Monoclonal Rat Antibody ADRP(B-6); Santa Cruz—Interprise^®^, Paulínia, SP, Brazil) following the protocol of the dip-coating technique; this involves immersion, start up, deposition, drainage, and evaporation steps based on a previous study [22]. For this, the implants were isolated and roughly coated with Scl-Ab (10 mg/µL) by immersing the implant in a mixture of 10 µL of Scl-Ab and 990 µL of distilled water (SS Plus do Brazil Ltda., Maringá, PR, Brazil), diluted in 100 µL of dimethyl sulfoxide (ACS Reagent, ≥99.9; Sigma-Aldrich, St. Louis, MO, USA). Subsequently, a dental air syringe connected to an air compressor was gently activated to blow air over the implant’s surface, ensuring a homogeneous coating. This procedure was repeated five times for each implant until the entire solution was finely coated onto the implant surface. Before surgery, the implants were sterilized using UV light for 20 min.

### 2.6. Implant Surgery

The animals were fasted for eight hours before the surgical procedure and anesthetized with a combination of 5 mg/kg of xylazine (Xylazine, Coopers Brazil Ltda., Cotia, SP, Brazil) and 50 mg/kg of ketamine (Fort Dodge, Animal Health Ltda., Campinas, SP, Brazil). They also received 0.3 mL/kg mepivacaine hydrochloride (Scandicaïne 2% with adrenaline 1:100.000; Septodont, Saint-Maur-des-Fossés, France) as a local anesthetic and to ensure hemostasis in the surgical field. The medial portion of the right and left tibia was shaved, and the region to be incised was disinfected with polyvinylpyrrolidone iodine degermante (PVPI 10%; Riodeine Degermante, Rioquímica, São José do Rio Preto, SP, Brazil) combined with topical PVPI 10%. An incision of 1.5 cm was made in the metaphyseal region of both tibias using a n^o^ 15 blade (Feather Industries, Tokyo, Japan). Then, the soft tissue was fully dissected and retracted using periosteal elevators, exposing the bone. For the placement of the implants, drilling was performed using a 1.6 mm diameter spiral drill bit mounted on an electric motor (BLM 600^®^; Driller, São Paulo, SP, Brazil) at a speed of 1000 rpm under irrigation with 0.9% of sodium chloride solution (Fisiológico^®^ Biosynthetic Laboratories Ltda., Ribeirão Preto, SP, Brazil) and a contra angle with a 20:1 reduction (3624N 1:4 Head 67RIC 1:4; KaVo^®^ Dental GmbH, Biberach, Germany), ensuring locking and initial stability. Each animal received two implants, one in each tibial metaphysis. The tissues were sutured in layers using absorbable suture (Polyglactin 910 4.0, Ethicon^®^; Johnson & Johnson, São José dos Campos, SP, Brazil) with continuous sutures in the deep layer and monofilament suture (Nylon 5.0, Ethicon^®^; Johnson & Johnson, São José dos Campos, SP, Brazil). In the immediate postoperative period, each animal received a single intramuscular dose of 0.2 mL of penicillin G-benzathine (Pentabiótico^®^, Veterinário Pequeno Porte, Fort Dodge Saúde Animal Ltda., Campinas, SP, Brazil).

### 2.7. Euthanasia

The euthanasia of the animals involved an overdose of 150 mg/kg 2.5% sodium thiopental (Fort Dodge Saúde Animal Ltda., Campinas, SP, Brazil) with 10 mg/kg 2% lidocaine (Laboratório Bravet Ltda., Rio de Janeiro, RJ, Brazil). Clinically, all the groups showed no signs of inflammation or infection. Therefore, no exclusions were made in any group.

### 2.8. Biomechanical Analysis (Removal Torque)

The right tibias were accessed to expose the implants and perform removal torque using a digital torque meter (TQ-680 Model; Instrutherm, São Paulo, SP, Brazil) coupled to the implant mount (Neodent, Curitiba, Paraná, Brazil) and adapted to the internal hexagon. The counterclockwise movement was applied, increasing the reverse torque until the bone–implant interface was broken. At this moment, the torque meter recorded the maximum value in Newton centimeters (Ncm) [23].

### 2.9. Real-Time Reverse Transcription Polymerase Chain Reaction (RT-PCR)

After performing reverse torque, the same tibias were used to evaluate gene expression related to bone repair through RT-PCR analysis. To preserve the integrity of the bone–implant interface, at least 0.5 cm of the bone surrounding each peri-implant area was collected. The samples were washed with a phosphate buffer solution, frozen with liquid nitrogen for total RNA extraction using Trizol reagent (Life Technologies Invitrogen, Carlsbad, CA, USA), and stored in a freezer at −80 °C. After assessing RNA integrity, purity, and concentration, cDNA synthesis was carried out using 1 µg of RNA and reverse transcription with M-MLV RT (Promega Corporation, Madison, AL, USA). The synthesized cDNA was then analyzed using TaqMan Fast Advanced Mastermix (Applied Biosystems, Waltham, MA, USA) in a 96-well PCR plate (Thermo Fisher Scientific, Waltham, MA, USA) to detect the gene expression of osteoprotegerin (OPG), the receptor activator of nuclear factor kappa-B ligand (RANKL), bone sialoprotein (IBSP), osteocalcin (OCN), and alkaline phosphatase (ALP). RT-PCR was conducted on the Step One Plus (Applied Biosystems, Waltham, MA, USA) under the following conditions: 50 °C (2 min) and 95 °C (10 min), followed by 40 cycles of 95 °C (15 s) and 60 °C (1 min), and then the dissociation curve. The relative gene expression was calculated against the mitochondrial ribosomal protein following the ΔΔCT method. The assay was performed in quadruplicate [3]. The TaqMan probes and primer sequences for RT-PCR are listed in Table 1.

### 2.10. Microcomputed Tomography (Micro-CT)

The left tibias were scanned by microcomputed tomography (SkyScan 1272 2003 Model; Bruker, Aatselaar, Belgium) with 9 μm of thick sections (50 kV and 500 μ) using a filter of aluminum and copper (Al 0.5 + Cu 0.038) with a rotation step of 0.3 mm. The images from the X-ray projections on the samples were reconstructed, defining the region of interest (ROI) using NRecon Software (SkyScan Version 1.6.6.0; Leuven, Belgium). In Data Viewer Software (SkyScan Version 1.4.4; Leuven, Belgium), the images were reconstructed to standardize the positioning for all samples, allowing observation in the planes: transversal, longitudinal, and sagittal. After that, using CTAn Software (SkyScan Version 1.12.4.0; Leuven, Belgium), an area around the implant (ROI) was defined and delimited by 0.5 mm around the entire implant. This area was defined as the total area (0.5 mm margin around the implants—ROI 4.5 mm × 3.2 mm). CTAn Software (SkyScan Version 1.12.4.0; Leuven, Belgium) analyzed and measured the image according to grayscale. The threshold used in the analysis was 25–90 grayscale, allowing the calculation of the percentage of bone volume (BV/TV) formed around the implants. Parameters characterizing the bone trabeculae were also evaluated, such as trabecular thickness (Tb.Th), number (Tb.N), and separation (Tb.Sp) between bone trabeculae, as well as connectivity density (Conn. Density) and surface intersection (IS) [24,25].

### 2.11. Laser Confocal Microscopy

The same samples of micro-CT were processed for dehydration in concentrations of ethanol (70%, 90%, and 100%). The solution was changed every five days, and the samples were incubated in an orbital shaker (KLine CT-150^®^; Cientec-Laboratory Equipment, Piracicaba, SP, Brazil) for four hours each day. Once dehydrated, the samples were embedded in methyl methacrylate (MMA)-based resin (Technovit^®^ 400; Kulzer Technik, Wehrheim, Germany) and taken in an oven at 37 °C for 5 days to allow the resin to thermo-polymerize. After that, the specimens were reduced using a maxi-cut drill mounted on a bench motor (Strong 210; Kota Indústria e Comércio, São Paulo, SP, Brazil). Then, progressive grinding was performed using a polisher machine (AutoMet^®^ 250 Pro Grinder-Polisher; Buehler, Lake Bluff, IL, USA) until the sections reached a thickness of 80 µm. The measurements were taken with a digital caliper (Mitutoyo, Pompeia, SP, Brazil), and the final sections were placed on a histological slide and sealed with a coverslip. The glass slides were submitted to laser confocal microscopy using a 10x objective lens (Stellaris 5; Leica Microsystems, Wetzlar, Germany). The images began from the onset of fluorescence, representing the beginning of calcification (calcium precipitation in the organic matrix). These images had a dimension of 1 × 1 mm^2^ and corresponded to optical sections of 512 by 512 pixels. The sections of 2 μm were scanned for 2.5 min. Thus, 28 cuts were obtained for every 56 μm of scanning. The barrier filters used were BP 530/30 nm and 590 LP, combined with the activation of the “double dichroic” 488/568 nm, and the photomultiplier was set to 534 for calcein and 357 for alizarin. Codes 534 and 357 nm represented the filters that allowed the visualization of fluorochromes. One of the filters enabled the visualization of calcein (blue filter), and the other filter, alizarin (green filter). The peri-implant bone presented two overlapping fluorochromes (calcein and alizarin), showing the conversion from old bone to new bone. These images were saved in TIFF format and transferred to ImageJ Software (Processing Software and Image Analysis Version 1.54p; Hamilton, ON, Canada) through the “color threshold” tool. Each image was standardized according to tone, saturation, and brightness. First, calcein was highlighted, and the “measure” tool was used to provide the area in μm^2^. The same procedure was performed for alizarin, obtaining data on the dynamics of the alveolar bone. Bone turnover was represented by the difference between old bone (green) and new bone (red). From the quantification of the fluorochrome areas, the calculation of the active mineralization surface, that is, the area of precipitation of the last injected fluorochrome (alizarin), was performed. The distance between the fluorochrome bands was measured considering the number of days between each injection; it was possible to calculate the mineral deposition rate. Therefore, the parameters analyzed were fluorochrome dynamics, the mineral apposition rate (MAR), neoformed bone area (NBA), and bone–implant contact (BIC) [26].

### 2.12. Statistical Analysis

For statistical analysis, GraphPad Prisma Software 8.1.1 was used. Homoscedasticity analysis was performed using the Shapiro–Wilk test. After performing the two-way ANOVA for all analyses, Tukey’s post hoc test was used to compare group means. The letters (A, B, C, D, and E) are used to indicate statistical differences between groups, where groups with the same letter do not differ significantly, while groups with different letters show statistically significant differences based on a significance level of *p* < 0.05.

## 3. Results

### 3.1. Removal Torque

**Figure 2:**
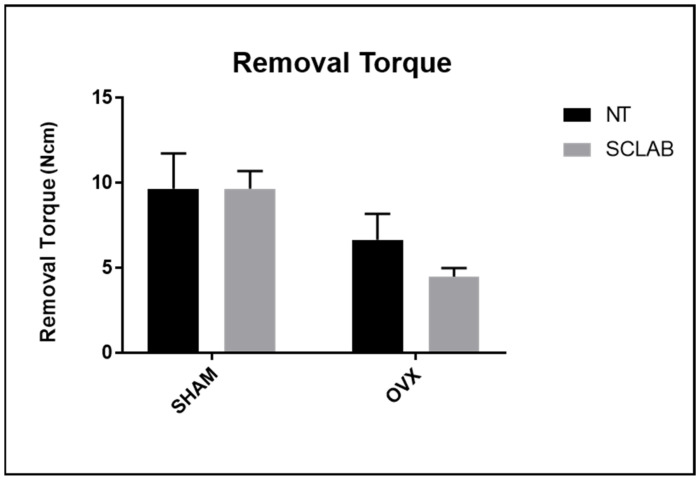
The reverse torque was conducted until the rotation of the implant from the bone, and the maximum recorded value in Ncm was noted for the groups that received or did not receive surface treatment with Scl-Ab. No statistically significant difference exists between the groups in all parameters (Tukey, *p* > 0.05).

Biomechanical analysis obtained the average value for SHAM NT, 8.25 Ncm; OVX NT, 6 Ncm; SHAM Scl-Ab, 9.15 Ncm; and OVX Scl-Ab, 6.60 Ncm. However, there were no statistically significant differences among the groups (*p* > 0.05) (Figure 2).

### 3.2. RT-PCR

**Figure 3:**
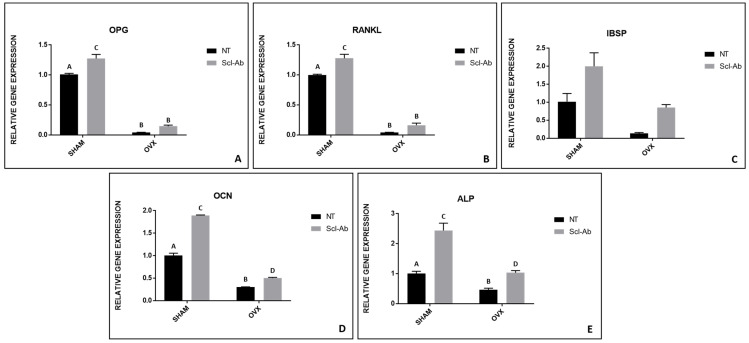
Relative gene expression analysis from the RT-PCR of all groups. (**A**) OPG—osteoprotegerin; (**B**) RANKL—receptor activator of nuclear factor kappa-B ligand; (**C**) IBSP—bone sialoprotein; (**D**) OCN—osteocalcin; (**E**) ALP—alkaline phosphatase. Significantly statistical differences are indicated with different letters (Tukey, *p* < 0.05), while groups with the same letter do not differ significantly.

For all parameters evaluated in the RT-PCR, the SHAM group was considered the standard, receiving a fixed value of 1, and the others were assessed relative to this reference. For the relative gene expression of OPG, higher values were observed in the SHAM Scl-Ab group (1.27), followed by SHAM NT (1.00). The OVX NT and OVX Scl-Ab groups showed the lowest results, with 0.14 and 0.04, respectively. In the comparison between the different groups, all of the results were statistically significant (Tukey, *p* < 0.05) (Figure 3A). For RANKL, the relative gene expressions of the SHAM NT, SHAM Scl-Ab, OVX NT, and OVX Scl-Ab groups were 0.99, 1.27, 0.04, and 0.16, respectively. Statistically significant differences were observed in the comparison between all groups (Tukey, *p* < 0.05) (Figure 3B). The relative gene expression of IBSP showed similar values for the SHAM NT (1.00) and OVX Scl-Ab (0.85) groups, which did not show statistically significant differences in the comparisons (ANOVA, *p* > 0.05) (Figure 3C). For OCN, the relative gene expression of all groups showed statistically significant differences in comparisons (Tukey, *p* < 0.05) with the following values: SHAM NT (1.00), SHAM Scl-Ab (1.89), OVX NT (0.29), and OVX Scl-Ab (0.50) (Figure 3D). Similar to OCN, a relevant pattern was found in the comparison between the groups for ALP, where the highest result was found in the SHAM Scl-Ab group (2.43). The lowest result was observed in the OVX NT group (0.45). However, more similar results were found for SHAM NT and OVX Scl-Ab, with values of 1.00 and 1.03, respectively (SHAM NT and OVX Scl-Ab vs. OVX NT vs. SHAM Scl-Ab: Tukey, *p* < 0.05) (Figure 3E). Statistically significant differences between groups are indicated by different letters (Tukey, *p* < 0.05), while groups sharing the same letter do not differ significantly (Tukey, *p* > 0.05) (Figure 3).

### 3.3. Micro-CT

**Figure 4:**
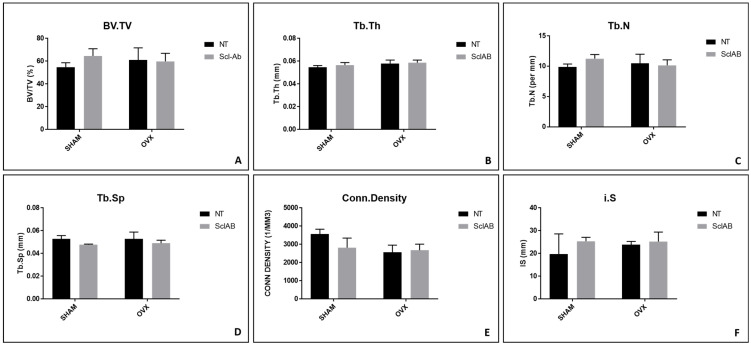
Microcomputed tomography. Morphological parameters were evaluated: (**A**) BV/TV—percentage of bone volume; (**B**) Tb.Th—trabecular thickness; (**C**) Tb.N—number of trabeculae; (**D**) Tb.Sp—separation of trabeculae; (**E**) Conn. Density—connectivity density; (**F**) i.S—surface intersection. No statistically significant difference exists between the groups in all parameters (Tukey, *p* > 0.05).

**Figure 5:**
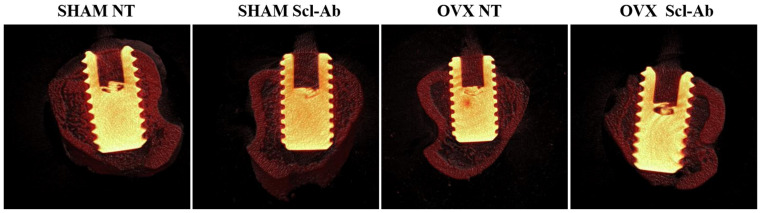
The three-dimensional image of peri-implant bone repair area of micro-CT using CTvox Software (SkyScan 2.7) representing all four groups: SHAM NT, SHAM Scl-Ab, OVX NT, and OVX Scl-Ab, respectively.

In the evaluation of BV/TV, the following results were found for the SHAM NT, SHAM Scl-Ab, OVX NT, and OVX Scl-Ab groups: 54.75%, 64.59%, 61.12%, and 59.84%, respectively (*p* > 0.05) (Figure 4A). Regarding the peri-implant trabecular bone, Tb.Th showed similar results across all SHAM NT and OVX NT groups with the following measurements: SHAM NT (0.054 mm), SHAM Scl-Ab (0.05 mm), OVX NT (0.05 mm), and OVX Scl-Ab (0.05 mm), with no statistically significant differences among the groups (*p* > 0.05) (Figure 4B). For Tb.N, the averages were 9.89/mm for OVX NT, 10.47/mm for SHAM NT, 11.26/mm for SHAM Scl-Ab, and 10.15/mm for OVX Scl-Ab (*p* > 0.05) (Figure 4C). The pattern observed for Tb.Sp showed similar results among all groups: SHAM (0.05 mm), SHAM Scl-Ab (0.04 mm), OVX NT (0.05 mm), and OVX Scl-Ab (0.04 mm) (*p* > 0.05) (Figure 4D). Regarding Conn. Density, the values were 3566.84/mm^3^ (SHAM NT), 2818.01/mm^3^ (SHAM Scl-Ab), 2561.76/mm^3^ (OVX NT), and 2678.47/mm^3^ (OVX Scl-Ab) (*p* > 0.05) (Figure 4E). For the IS, a relatively similar area was assessed among the groups: 19.64 mm^3^ (SHAM NT), 25.37 mm^3^ (SHAM Scl-Ab), 23.86 mm^3^ (OVX NT), and 25.21 mm^3^ (OVX Scl-Ab), with no statistically significant differences among the groups (*p* > 0.05) (Figure 4F). The three-dimensional image of peri-implant bone repair can be observed in Figure 5.

### 3.4. Laser Confocal Microscopy

**Figure 6:**
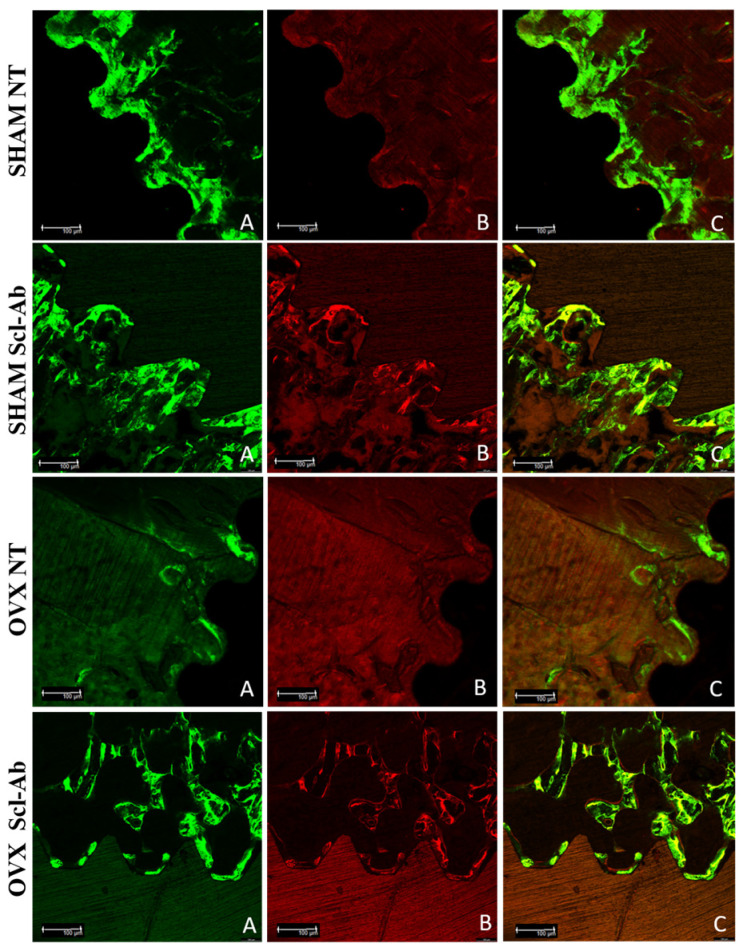
Photomicrographs were obtained using laser confocal microscopy in all four groups: SHAM NT, SHAM Scl-Ab, OVX NT, and OVX Scl-Ab, respectively. (**A**) Bone area marked with calcein; (**B**) bone area marked with alizarin; (**C**) overlapping images of both fluorochromes. Scale: 100 µm.

**Figure 7:**
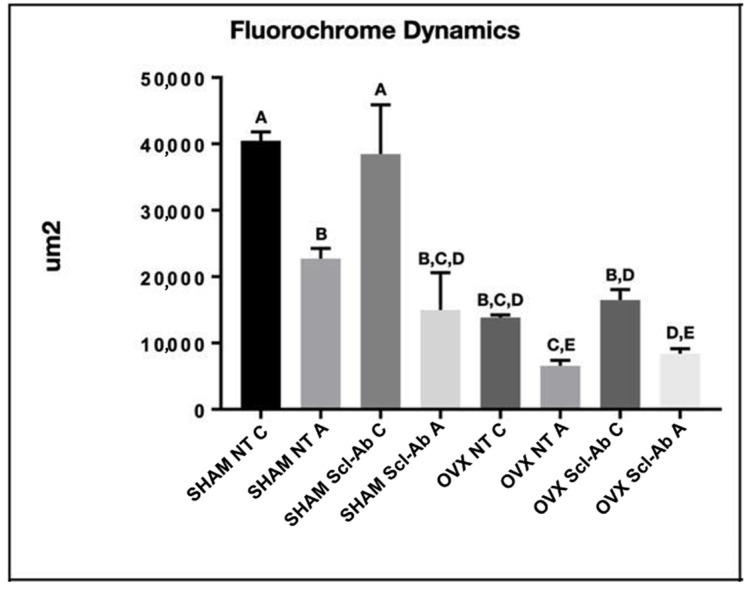
Quantification of laser confocal microscopy. Fluorochrome dynamics. The highest values indicated the statistically significant difference between SHAM NT (calcein) and SHAM Scl-Ab (calcein) (Tukey, *p* < 0.05). Significantly statistical differences are indicated with different letters (Tukey, *p* < 0.05), while groups with the same letter do not differ significantly.

**Figure 8:**
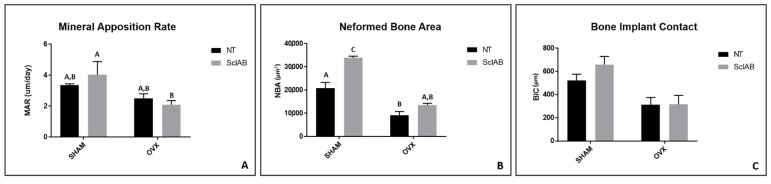
Quantification of laser confocal microscopy. (**A**) MAR—mineral apposition rate; (**B**) NBA—neoformed bone area; (**C**) BIC—bone–implant contact. Significantly statistical differences are indicated with different letters (Tukey, *p* < 0.05), while groups with the same letter do not differ significantly.

The fluorochromes labeled the calcium matrix with calcein (green) and alizarin (red), demonstrating the dynamics of the peri-implant bone during defect repair in the experimental groups. This allowed for assessments of bone dynamics, daily calcium deposition, and the area of new bone formation (Figure 6). In the comparison between different groups, the average results showed the highest numerical values for the SHAM NT (calcein) and SHAM Scl-Ab (calcein) groups, with statistically significant differences compared to the other groups (Tukey, *p* < 0.05) (Figure 7). For the MAR that evaluates the mineral deposition of calcium on the bone matrix, between the intervals of calcein and alizarin applications, the results were 3.37 µm (SHAM NT), 4.03 µm (SHAM Scl-Ab), 2.50 µm (OVX NT), and 2.10 µm (OVX Scl-Ab). Statistical analysis revealed a pattern showing increased mineral apposition due to topical Scl-Ab only in the SHAM NT group, where no statistically significant difference was observed when compared to the OVX NT and OVX Scl-Ab groups (Tukey, *p* < 0.05) (Figure 8A). Regarding NBA assessing the amount of new bone that was formed per area after the bone repair process, the overlay images of old and new bone showed more favorable results in peri-implant new bone formation in healthy rats treated with topical Scl-Ab (SHAM Scl-Ab—33.88 µm^2^), which showed a statistically significant difference compared to the other groups (Tukey, *p* < 0.05). Additionally, a significant improvement in new bone formation was noted in the OVX NT group (9.22 µm^2^) when Scl-Ab was added to the surface (OVX Scl-Ab—13.49 µm^2^), also showing statistically significant results in this comparison (Tukey, *p* = 0.0411) (Figure 8B). Because BIC is a measure used to evaluate the integration of the implant with the surrounding bone, the results for SHAM NT (521.10 µm) were lower than for SHAM Scl-Ab (659.60 µm), but there was no statistically significant difference between these two groups (ANOVA, *p* > 0.05). The same pattern was observed for OVX NT (312.60 µm) compared to OVX Scl-Ab (314.80 µm), where the functionalization with Scl-Ab improved the result, but no statistical significance was found between these two groups (ANOVA, *p* > 0.05). However, there was statistical significance in the comparison of SHAM NT and SHAM Scl-Ab with both OVX NT and OVX Scl-Ab (Tukey, *p* < 0.05) (Figure 8C). Statistically significant differences between groups are indicated by different letters (Tukey, *p* < 0.05), while groups sharing the same letter do not differ significantly (Tukey, *p* > 0.05) (Figure 7 and Figure 8).

## 4. Discussion

The success of oral implants is linked to their ability to achieve osseointegration, making the geometry and surface topography of implants critical factors for both short- and long-term outcomes [27]. In this context, the development of new surface treatments aimed at modifying implant topography and chemistry presents significant challenges. This study investigates the quantitative effects of a surface functionalized with a local anti-sclerostin bone anabolic drug (Scl-Ab) on implants. The evaluation focuses on bone tissue microarchitecture parameters and relative gene expression, particularly in healthy and ovariectomized rats.

In recent years, in vitro and in vivo studies have been conducted to assess the effectiveness of implant surface modifications, highlighting properties that enhance cellular activity [28] and promote bone consolidation and apposition [29,30]. Some examples of surface functionalization include the application of platelet-rich fibrin [31] and rhBMP-7 combined with nanometric hydroxyapatite to achieve improved osseointegration [32].

The anti-sclerostin anabolic drug (Scl-Ab) is available under the trade name Romosozumab [7]. This drug acts directly on the WNT pathway by inhibiting sclerostin, improving the bone repair process [33]. Its action leads to the binding of WNT to the β-catenin pathway, phosphorylating β-catenin and thus promoting bone formation [33,34]. This is an important reason to evaluate this drug in the dental implant model, as it has already been demonstrated that the Wnt/β-catenin pathway is involved in better reparative responses of bone tissue in post-extraction sockets and around implants [35,36,37].

Current studies have shown that Scl-Ab can improve implant osseointegration by promoting greater bone–implant contact extension. Still, nothing has been found regarding the peri-implant regeneration process when functionalizing implants with their local application [19,38,39]. The incentive for local testing through the availability of a monoclonal antibody (Scl-Ab) that resembles the drug treatment of Romosozumab exists as a strategy to assess the reparative response, which is why the present study presents the results of ongoing testing in this manner [39].

The biomechanical analysis presented reveals significant changes between the studied groups; the mean torsional resistance value for the SHAM NT group was 8.25 Ncm, while for the OVX NT group, it was 6 Ncm, which demonstrates a reduction in torsional resistance after ovariectomy (OVX). This result is consistent with the literature, which often observes that ovariectomy can compromise bone biomechanics, leading to a decrease in strength and structural integrity [40].

The results obtained through micro-CT indicate that treatment with Scl-Ab positively impacted bone formation, especially in the group of healthy rats. However, the effect of Scl-Ab was insufficient to significantly attenuate bone formation in the OVX group. For the laser confocal microscopy analysis, bone dynamics were positively affected, where there was greater calcium deposition, new bone formation, and an increase in mineral apposition (MAR), in which there was a statistically significant difference between the SHAM Scl-Ab vs. OVX Scl-Ab groups. For the OVX group, treatment with Scl-Ab did not improve new bone formation, but there was no statistical difference compared to the OVX group without treatment. For the bone-to-implant contact (BIC) parameter, there was no significant difference between the treated and control groups, suggesting that Scl-Ab did not substantially influence bone adhesion to the implant.

Scl-Ab was shown to be effective in promoting bone regeneration under healthy conditions, as evidenced by increased calcium deposition and new bone formation in the SHAM Scl-Ab group but had a limited impact on ovariectomy-compromised bones. The absence of significant changes in bone microarchitecture parameters and peri-implant bone dynamics suggests that Scl-Ab, although promoting bone regeneration, does not substantially influence global bone structure in osteopenic models; as pointed out in the literature, the local application of anti-sclerostin has limitations regarding its potential effectiveness [41].

Although Scl-Ab had no influence in improving the removal torque and did not affect any micro-CT or confocal parameters, positive results were found in RT-PCR, especially for IBSP and ALP relative gene expression, where the highest result was in the SHAM Scl-Ab group. This shows that relative gene expression was present in the OVX group when Scl-Ab was added to the surface of their implants, leading to a stimulus similar to that in SHAM, thus drawing an analogy between the treated animal with estrogen deficiency and the untreated healthy one.

Low estrogen levels may impair bone repair around implants, especially in areas with bone defects [15]. It is important to explore therapies that use local drugs applied to the implant surface to improve peri-implant bone repair and reduce the risk of compromised osseointegration due to estrogen deficiency [24,42,43]. This condition may impair bone regeneration by inhibiting osteoblast differentiation, maturation, and matrix formation [43]. Systemic Scl-Ab has demonstrated efficacy in promoting bone regeneration, but studies investigating its local use are still lacking, especially in the context of peri-implant bone defects, where direct application could potentially improve outcomes [19,38].

Therefore, in this study, the estrogen deficiency model with ovariectomized rats is an additional challenge for the functionalization of the tested implants, as well as the possibility of evaluating the performance of the monoclonal antibody (Scl-Ab) locally at the repair site. For this reason, through the results of RT-PCR, we observed better functionalization performance with monoclonal antibody (Scl-Ab) in SHAM than in the OVX NT group. The results of this study should be interpreted in light of its limitations. The primary limitation is that this study lacks experimental support for the potential mechanisms of action of the medication through in vitro experiments. In addition, no analysis was conducted to characterize the implant surface. Therefore, evaluating changes in surface wettability and roughness in future studies is crucial, as these properties may significantly influence the biological outcomes.

## 5. Conclusions

The results indicate that the functionalization of implants with an anti-sclerostin monoclonal antibody (Scl-Ab) can improve peri-implant bone repair, especially in healthy rats. Although the results are promising, this study has limitations, such as the lack of in vitro experiments and the absence of analysis of the implant surface properties, which are fundamental to the biological results. To confirm the therapeutic benefits of local Scl-Ab, further research is needed, including the evaluation of cellular mechanisms and implant surface characteristics.

## Figures and Tables

**Table 1:** TaqMan probes and primer sequences for RT-PCR analysis.

Gene	Gene Reference	Gene Code	Forward Primer, 5′→3′	Reverse Primer, 5′→3′
OPG	NM_057149.2	Rn00563499_m1	GCACTCCTGGTGTTCTTGGA	TTTGGTCCCAGGCAAACTGT
RANKL	NM_057149.1	Rn00589289_m1	CGAGCGCAGATGGATCCTAA	GAGCCACGAACCTTCCATCA
IBSP	NM_012587.2	Rn00561414_m1	GTACCGGCCACGCTACTTTC	ATCTCCAGCCTTCTTGGGTAGC
OCN	NM_013414.1	Rn00566386_g1	CTCTGAGTCTGACAAAGCCTTCAT	GTAGCGCCGGAGTCTATTCA
ALP	NM_031144.3	Rn00564931_m1	GAGGAACGGATCTCGGGGTA	ATGAGTTGGTAAGGCAGGGTC

## Data Availability

The authors will make the raw data supporting this article’s conclusions available upon request.
